# Infections due to dysregulated immunity: an emerging complication of cancer immunotherapy

**DOI:** 10.1136/thoraxjnl-2021-217260

**Published:** 2021-10-04

**Authors:** Tommaso Morelli, Kohei Fujita, Gil Redelman-Sidi, Paul T Elkington

**Affiliations:** 1 NIHR Biomedical Research Centre, School of Clinical and Experimental Sciences, Faculty of Medicine, University of Southampton, Southampton, UK; 2 Respiratory Medicine, National Hospital Organisation Kyoto Medical Center, Kyoto, Japan; 3 Division of Infectious Diseases, Memorial Sloan Kettering Cancer Center, New York, New York, USA; 4 Institute for Life Sciences, University of Southampton, Southampton, UK

**Keywords:** opportunist lung infections, lung cancer, lung cancer chemotherapy, tuberculosis, aspergillus lung disease, viral infection, bacterial infection

## Abstract

Immune checkpoint inhibitors (ICIs) have revolutionised cancer treatment. However, immune-related adverse events (irAEs) are a common side effect which can mimic infection. Additionally, treatment of irAEs with corticosteroids and other immunosuppressant agents can lead to opportunistic infection, which we have classed as immunotherapy infections due to immunosuppression. However, emerging reports demonstrate that some infections can be precipitated by ICIs in the absence of immunosuppressive treatment, in contrast to the majority of reported cases. These infections are characterised by a dysregulated inflammatory immune response, and so we propose they are described as immunotherapy infections due to dysregulated immunity. This review summarises the rapidly emerging evidence of these phenomena and proposes a new framework for considering infection in the context of cancer immunotherapy.

## Introduction

In 1957, Macfarlane Burnet wrote ‘The failure in cancer is due not to any weakness of the organism, but to a change in the character of the cells, rendering them in one way or another insusceptible to the normal control’.^1^ Subsequently, evading immune destruction was identified as a critical hallmark of cancer.^2^ The 2018 Nobel Prize was awarded to James Allison and Tasuku Honjo for the discovery that cancer cells could exploit programmed cell death protein 1 (PD-1) and cytotoxic T-lymphocyte associated protein 4 (CTLA-4) signalling to avoid immune destruction.^3^

PD-1 is an immune checkpoint molecule expressed on effector T cells and binds to PD-L1 and PD-L2 on antigen-presenting cells (APCs). Tumour cells can also express PD-L1 as a method of evading immune destruction, whereby PD-1/PD-L1 signalling supresses effector T-cell priming and proliferation. CTLA-4 is another coinhibitory receptor expressed on effector T cells. CTLA-4 competes with the costimulatory receptor CD28 on effector T cells for binding with activation ligands CD80/86, found on APCs. CD28 binding to CD80/86 is essential for T-cell receptor signalling, and hence CTLA-4, which binds to CD80/86 with greater affinity, effectively prevents effector T-cell activation. CTLA-4 and PD-1 are expressed on effector T cells following chronic, sustained antigen exposure.^4–6^

PD-1 and CTLA-4 signalling thus acts as a ‘brake’ to balance and prevent an over-exuberant damaging immune response, for example, in chronic infection. PD-1/PD-L1 signalling can be inhibited using monoclonal antibodies against PD-1 (eg, pembrolizumab and nivolumab) and PD-L1 (eg, durvalumab, atezolizumab and avelumab). CTLA-4 signalling can similarly be blocked using monoclonal antibodies such as ipilimumab and tremelimumab. Immune checkpoint inhibitors (ICIs) have revolutionised cancer treatment, with improved survival demonstrated in many forms of cancer, including lung cancer, melanoma, colorectal cancer, hepatocellular carcinoma, renal cell carcinoma, bladder cancer, Merkel cell carcinoma and Hodgkin’s lymphoma.^4^ ICIs require close monitoring as they are associated with autoimmune phenomena known as immune-related adverse events (irAEs).^4^

### Immune-related adverse events

IrAEs are a common side effect of ICIs, with an incidence between 54% and 76% according to a metanalysis of trial data.^7^ IrAEs can affect any body system, and management often involves treatment with glucocorticoids such as prednisolone and other immunosuppression such as anti-TNF therapy for steroid refractory cases. From the pulmonary perspective, immune-related pneumonitis occurs more commonly with PD-1 blockade and has several radiological presentations including cryptogenic organising pneumonia, non-specific interstitial pneumonia, hypersensitivity pneumonitis, acute interstitial pneumonia and pulmonary sarcoid reactions.^5^ Combination ICI therapy appears to increase the risk of irAEs.^8^ The pathogenesis of irAEs may be mediated by T-cell autoimmunity, impaired regulatory T-cell (Treg) function, TH17 helper T cells and T cell-mediated autoantibodies.^4^ Notably, irAEs can mimic infection.^9^ Recently, it has been hypothesised that immune checkpoint blockade may cause increased immune recognition of commensal bacteria, such as gut microbiota, perhaps via attenuated Treg function, and this immune recognition of commensal bacteria can cause expansion of TH17 cells that have the potential to migrate and potentially cause irAEs.^10^

### Consequences of natural immune checkpoint loss of function

Immune checkpoints help prevent activity against self. Consequently, polymorphisms in CTLA-4 and PD-1 are associated with autoimmune sequalae such as Addison’s disease, coeliac disease, Graves’ disease, type 1 diabetes mellitus, myasthenia gravis, rheumatoid arthritis and systemic lupus erythematosus.^4 6^

In addition, genetic mutations in these receptors predict that pharmacological targeting of immune checkpoints may have infectious complications. CTLA-4 haploinsufficiency with autoimmune sequelae (CHAI) and lipopolysaccharide-responsive and beige-like anchor protein (LRBA) deficiency with autoantibodies, Treg defects, autoimmune infiltration and enteropathy (LATAIE) are human genetic disorders associated with deficient CTLA-4 expression and function. CHAI and LATAIE manifest with multiorgan lymphocytic infiltration, T_reg_ defects and autoantibody production, and are also associated with recurrent infections, particularly respiratory infections.^11^ A review of the phenotype of patients with LATAIE revealed 71% suffered from recurrent infections (upper respiratory tract, lower respiratory tract and urinary infections). Reported bacterial infections included a range of pathogens, such as *Escherichia coli*, *Klebsiella pneumoniae*, *Haemophilus influenzae*, *Pseudomonas aeruginosa*, *Campylobacter* and *Staphylococcus aureus*. Reported viral infections include cytomegalovirus (CMV), adenovirus, norovirus and varicella zoster, and *Candida* infections are also described.^12^ Although these genetically determined phenotypes have more severe manifestations than CTLA-4-associated irAEs, they provide some insight into the natural consequences of loss of CTLA-4 function. Interestingly CTLA-4 mutations have also been associated with more severe TB infection in an African population study.^13^ With respect to PD-1/PD-L1, PD-1-deficient mice are highly susceptible to TB infection, 14 15 and a case of inherited PD-1 deficiency associating with TB has very recently been reported.^16^

Given the emerging evidence of increased infection in patients treated with ICIs and evidence that immune checkpoint deficiency can be associated with recurrent infections, we were interested in evaluating the infectious sequelae of ICI therapy. Furthermore, we aimed to characterise the pattern of these infectious complications to inform future clinical management strategies and the associated research agenda.

### Literature search

**Figure 1:**
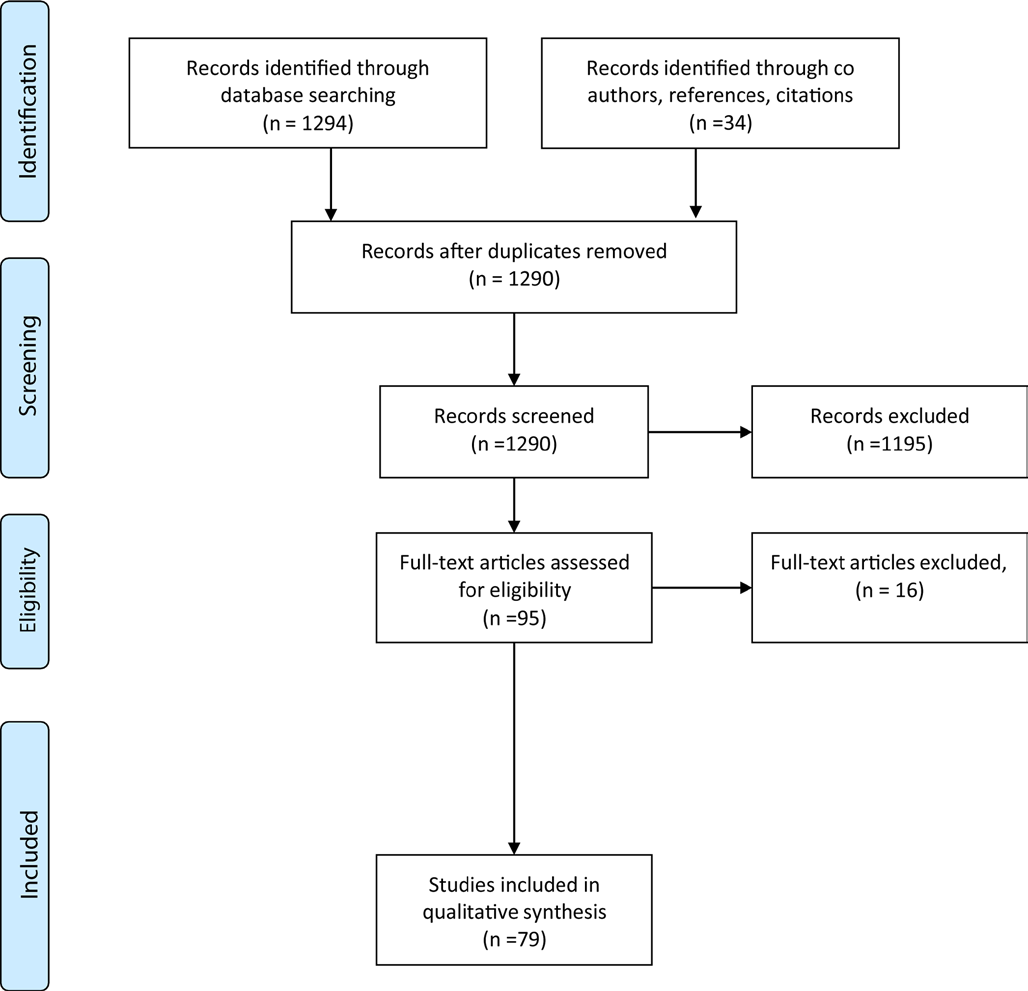
Preferred Reporting Items for Systematic Reviews and Meta-Analyses (PRISMA) chart.

We performed a literature search to identify reports of adult patients with cancer who were found to have developed infections after ICI initiation. The search protocol was registered on PROSPERO (CRD 4202141634, see online supplemental appendices 1–2). We searched Medline (Ovid 1996–week 4 February 2021) using advanced search, for the following keywords: (“Infection” or “Infectious Disease”) and (“Immune checkpoint inhibitor” or “PD-1” or “PD-L1” or “CTLA-4”). Additional limits were placed to include articles relevant to humans and those published in the English language. This search yielded 1294 results. Thirty-four additional relevant studies were identified through coauthor suggestion, references and citations. After screening for suitability by viewing the title, abstract, introduction and conclusion, and removing duplicates, 95 full-text articles were assessed. Seventy-nine studies were included for analysis (figure 1). Information on cancer type, ICI used, concurrent immunosuppression and available descriptive statistics was collected. Online supplemental table S1 summarises the studies of immunotherapy-associated infection.

10.1136/thoraxjnl-2021-217260.supp1 Supplementary data

### Prospective randomised clinical trial data

Trial data have not demonstrated a significantly increased rate or risk of infection following ICI treatment.^17^ A review of phase I trial data revealed infection-related adverse events in 18% of patients, and the OR of infection was not statistically different from molecular targeted agents for cancer.^18^ One study of patients with non-small cell lung cancer (NSCLC) treated with pembrolizumab reported complications of pneumonia (1.5%), lung infection (0.3%), oral candidiasis (0.3%) and urinary tract infection (0.3%).^19^ Another randomised controlled trial (RCT) reported a case of pneumonia and severe varicella zoster infection following nivolumab for NSCLC.^20^ Eight cases of drug-related infection, including one severe varicella zoster infection, were reported in a nivolumab trial for melanoma.^21^ Sepsis following atezolizumab for bladder cancer has also been reported.^22^ However, while RCTs are the gold standard for determining treatment efficacy by minimising bias, they do not adequately assess all potential treatment harms, especially if infrequent.^23^ Typically, these only come to light in postmarketing surveillance when greater numbers of patients are treated.^24^ Additionally, while the total reported infections is small, there may be under-reporting in trials as causality to ICIs is not clearly established. Thus, analysis of other forms of data such as clinical observational data is required.

### Opportunistic infections associated with irAE treatment

The European Society of Clinical Microbiology and Infectious Diseases provided a consensus statement which suggested that ICIs do not intrinsically increase the risk of infection, but rather immunosuppressive therapy to treat irAEs can predispose to opportunistic infection.^17^ This followed a retrospective cohort review from Del Castillo *et al*, who analysed records 740 patients with melanoma who received ICIs over a 4-year period and recorded the number of infections requiring hospitalisation or antimicrobial treatment.^25^ Seven per cent of patients developed infection and 17% of these patients died. The vast majority (73.2%) of patients had CTLA-4 blockade with ipilimumab; 14.9% had PD-1 blockade, while 11.7% had combination ICI therapy. Eighty-five per cent of infections were bacterial, with 13/23 being pneumonia. Two cases of invasive pulmonary aspergillosis, three *Pneumocystis jirovecii* pneumonia, one candidemia and one strongyloidiasis case were reported. Risk factors for infection included corticosteroid administration (OR 7.71, p<0.0001), infliximab (OR 4.74, p<0.0001) and combined PD-1/CTLA-4 blockade (OR 3.26, p 0.0017).^25^

There are multiple case reports which have also highlighted instances of opportunistic infection following irAE immunosuppression with a variety of causative pathogens including *Aspergillus fumigatus*,^26–31^ *P. jirovecii*,^31–34^ John Cunningham (JC) virus,^35^ CMV^31 33 36–38^ and *Campylobacter* 39 (summarised in table 1). These reports highlight the need to have a low threshold of investigation for opportunistic infection following irAE treatment and consideration of *P. jirovecii* prophylaxis. In addition to prior immunosuppressive cancer treatments, comorbidities such as COPD in patients with lung cancer and the immunosuppressive tumour microenvironment are risk factors for infections.^40 41^

**Table 1:** Immunotherapy infections due to immunosuppression

Authors	Study types	ICI class	Immunosuppressive agents	Pathogen
Arriola *et al*, Del Castillo *et al*, Schwarz *et al*, Liu *et al*, Si *et al* 25 31–34	Case report, cohort	CTLA-4, PD-1, PD-L1	Corticosteroids, TNF-α antagonists, mycophenolate mofetil	*Pneumocystis jirovecii*
Lord *et al*, Kyi *et al*, Del Castillo *et al*, Gupta *et al*, Oltolini *et al*, Liu *et al*, Malek *et al*, Taima *et al* 25–31 128	Case report, cohort	CTLA-4, PD-1, PD-L1	Corticosteroids, TNF-α antagonists, rituximab, mycophenolate mofetil, tacrolimus, rapamycin	*Aspergillus fumigatus*
Del Castillo *et al*, Liu *et al* 25 31	Case report, cohort	CTLA-4, PD-1, PD-L1	Corticosteroids, TNF-α antagonists	*Candida albicans*
Uslu *et al*, Del Castillo *et al*, Furuta et al, Guengen et al, Oltolini *et al*, Schwarz *et al*, Liu *et al* 25 31 33 36–38	Case report, cohort	CTLA-4, PD-1	Corticosteroids, TNF-α antagonists, Mycophenolate mofetil	Cytomegalovirus
Martinot *et al* 35	Case report, unpublished pharmacovigilance registry data (WHO and Eudra-Vigilance)	CTLA-4 (PD-1 registry data)	Corticosteroids	JC polyoma virus
Del Castillo *et al*, Oltolini *et al*, Lee *et al*, Liu *et al* 25 31 39	Case report, cohort	CTLA-4, PD-1, PD-L1	Corticosteroids, TNF-α antagonists	Other bacteria including non-specified organism, bacteraemic sepsis, *Pseudomonas aeruginosa*, *Stenotrophomonas maltophilia*, *Corynebacterium striatu*, *Campylobacter*

CTLA-4, cytotoxic T-lymphocyte associated protein 4; ICI, immune checkpoint inhibitor; PD-1, programmed cell death protein 1.

### ICI-associated infection independent of immunosuppression

While the predominant infection risk post-ICI treatment is likely to be due to immunosuppression, there is mounting evidence of a second mechanism that leads to increased incidence of infection. For example, Fujita *et al* reviewed 167 records of patients who received nivolumab for NSCLC, and 19.2% (32 patients) were treated for infection.^42^ There were 33 infections; 78.1% were bacterial and included *Streptococcus pneumoniae*, *H. influenzae*, *K. pneumoniae,* methicillin-resistant *S. aureus*, methicillin-sensitive *S. aureus*, *Staphylococcus schleiferi*, *Mycobacterium tuberculosis* (Mtb) and other unknown presumed bacterial infection. Of the total infections, 6.3% were fungal and included *A. fumigatus* and *Candida albicans* infection, while 18.8% were viral and included varicella and influenza. Diabetes significantly increased the risk of infection (OR 3.61, p=0.028).^42^ Of note, this study exclusively analysed PD-1 blockade in the form of nivolumab. Unlike the Del Castillo *et al* study, there was no significant difference in the number of infections with use of corticosteroids or other immunosuppressive agents, suggesting an alternative mechanism.

Kanjanapan and Yip recently published a cohort study of 327 patients with primarily NSCLC (36%) and melanoma (47%).^43^ The majority (77%) of patients had PD-1/programmed cell death 1 ligand 1 (PD-L1) therapy; 9% had CTLA-4 therapy; and 14% had combination therapy. The rate of infection up to 12 months post immunotherapy was 27%; age was the only identified risk factor for infections post-ICI initiation (HR 1.73, p=0.04), while corticosteroid use and diabetes mellitus were not risk factors for post-ICI infection. The rate of infection in the pre-ICI period was 34%, but notably infectious episodes were defined through positive culture or PCR, potentially allowing inclusion of commensal colonisation such as *S. aureus* on the skin (55 identified in the pre-ICI period and defined as infection). Furthermore, given the long study period of 7 years, it is not possible to directly compare the rates of infection pre-ICI and post-ICI. However, there was still a significant number of infections in the post-ICI period, and corticosteroids were not a statistically significant risk factor.^43^

Furthermore, a recent study retrospectively evaluated infectious sequelae in 200 patients in a French registry treated with PD-1 (98.5%) and PD-L1 inhibitors (1.5%). Of these patients, 60% had melanoma, and 35.5% had NSCLC.^44^ Infections occurred in 18% between 19 and 132 days after ICI initiation, of which 58.3% were suspected pulmonary infection, which improved with antibiotics; 19.4% were skin infections; 19.4% were urinary tract infections; and 2.9% were gastrointestinal infections. There were no cases of *P. jirovecii* or other typical opportunistic infections. No association with corticosteroids, or other immunosuppressant medication, was reported in this study, again suggesting that immunosuppression was not the primary driver.^44^

**Figure 2:**
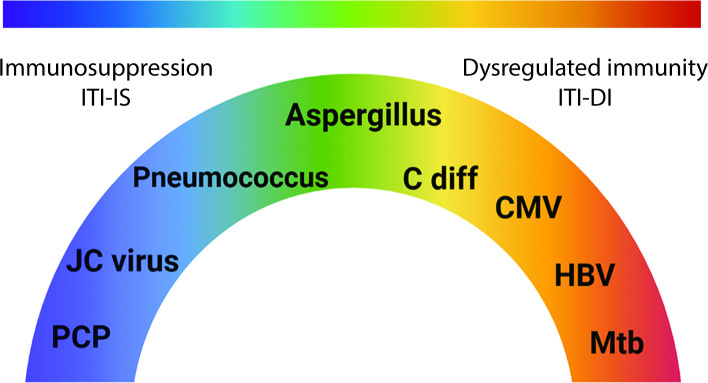
Spectrum of immunotherapy-associated infections. While immunosuppression alone can cause infectious complications, the dysregulated immunity that results from immune checkpoint inhibition can lead to different patterns of infection reactivation due to excessive inflammation, thereby resulting in a spectrum of disease phenotypes. ITI-DI, immunotherapy infections due to dysregulated immunity; ITI-IS, immunotherapy infections due to immunosuppression; Mtb, *Mycobacterium tuberculosis; C diff, Clostridium difficile; CMV, cytomegalovirus; HBV, hepatitis B virus*.

These more recent studies together suggest that other factors other than corticosteroids and immunosuppression may play a role in post-ICI infection. There are diverse reports of infection, in which it appears the hyperinflammatory dysregulated immunity associated with ICIs drives pathogenesis. We propose these can be characterised as immunotherapy infections due to dysregulated immunity (ITI-DI) to distinguish them from immunotherapy infections due to immunosuppression. ITI-DI may be considered an entirely different pathological mechanism whereby the excessive host immune response due to inhibition of immune checkpoints counterintuitively favours the pathogen (figure 2).

### Emerging examples of ITI-DI

#### Mtb and atypical mycobacterial infection (AMI)

**Figure 3:**
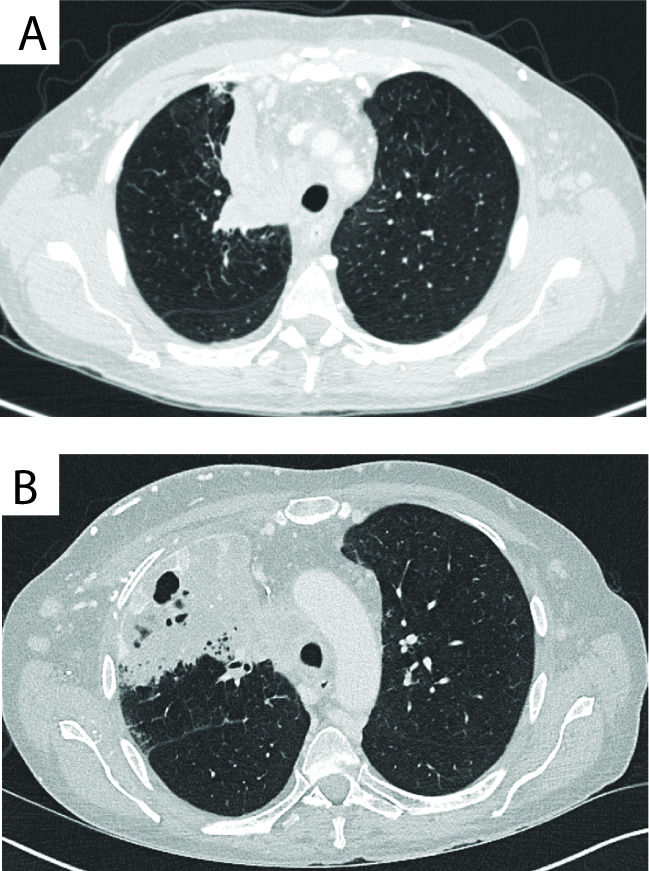
Development of pulmonary TB on triplet chemotherapy. Comparison of diagnostic CT scan performed 2 weeks before treatment (A) with the CT scan at the time of TB diagnosis (B) shows regression of the tumour with immune checkpoint inhibition but new consolidation with cavitation. Adapted from Crawley et al.^56^

TB reactivation was one of the first ICI-associated infections to be described. Currently, there are at least 19 case reports of Mtb reactivation following PD-1/PD-L1 blockade^45–60^ (figure 3 and online supplemental table S1). A review of the US Food and Drug Administration Adverse Events Reporting (FAERS) system for the incidence of Mtb revealed 72 cases of Mtb following PD-1/PD-L1 blockade. The reporting OR for Mtb infection with PD-1/PD-L1 inhibitors was 1.79 (95% CI 1.42 to 2.26) (p<0.0001),^61^ demonstrating increased risk of active TB. In addition, due to the clinical similarity in features of cancer progression and mycobacterial infection, it seems likely that cases are under-reported through underdiagnosis.^60^ Similarly, analysis of a single-centre cohort of 297 patients in Japan with lung cancer receiving treatment with pembrolizumab, nivolumab, atezolizumab or durvalumab revealed an incidence of Mtb reactivation of 1.7% developing 22–398 days after initiation of ICI treatment.^62^ This is higher than the incidence of Mtb reactivation in lung cancer reported in the 1980s, when generally higher doses of cytotoxic chemotherapy were used.^63^ Also, a single-centre cohort in South Korea identified three cases of Mtb and calculated an incidence rate of 394.4 per 100 000 person years, higher than the rate in the South Korean population in 2018 and previous studies that included patients with lung cancer.^64^ Finally, a single-centre cohort in Singapore showed that 4/191 (2.09%) of patients developed Mtb reactivation following PD-1/PD-L1 blockade.^65^

AMI has also (online supplemental file 1) been associated with immunotherapy. We found four cases of atypical mycobacterium infection following PD-1/PD-L1 immunotherapy in the absence of immunosuppression.^66 67^ Analysis of the FAERS database revealed 13 cases of AMI following PD-1/L1 blockade, similarly suggesting elevated risk of infection. The reporting OR for AMI was 5.49 (95% CI 3.15 to 9.55, p<0.0001).^61^

In terms of the underlying mechanism, transgenic murine studies have demonstrated that PD-1 knockout mice had increased susceptibility to Mtb infection, succumbing even more rapidly than interferon gamma (IFN-γ)-deficient mice.^14 15^ In a human 3D cell culture system, PD-1 blockade leads to excessive cytokine secretion, and tumour necrosis factor (TNF)-α may play a central role.^68^ Similarly, TB-infected macaques given anti-PD-1 antibodies develop worse disease, have higher bacterial loads as well as elevation of multiple inflammatory cytokines, compared with non-PD-1-treated macaques.^69^ Notably, these studies suggest PD-1 blockade negatively affects the host–Mtb interaction even in the absence of any immunosuppression, favouring pathogen proliferation over host control due to a dysregulated immune response.

These emerging clinical observations, animal modelling and advanced cell culture studies all suggest that disruption of the PD-1/PD-L1 axis results in hyperinflammatory conditions that favour mycobacterial growth. Consequently, diverse groups have suggested all patients should have interferon gamma release assay (IGRA) testing prior to ICI initiation, and there may be merit in serial IGRA testing before starting immunosuppressive therapies for irAEs.^48 70 71^ The evidence presented here further supports screening of patients for latent Mtb before starting anti-PD-1 treatment and investigation for mycobacterial infection when clinical features are suggestive, especially in high incidence settings. Notably, to date, reports of TB primarily centre on the PD-1/PD-L1 axis, and there are no cases of CTLA-4 inhibitor monotherapy causing Mtb reactivation.

### Hepatitis B virus

Hepatitis B-infected patients were excluded from the initial immunotherapy RCTs. Subsequently, there are multiple reports of hepatitis B reactivation, including cases where immunosuppression has been absent.^72–76^ Furthermore, Burns *et al* analysed the FAERS database for reported cases of hepatitis B and determined the reporting OR for reactivation with pembrolizumab to be 2.32 (95% CI 1.11 to 4.28, p=0.013),^77^ thus highlighting elevated risk of viral reactivation with pembrolizumab. Consistent with this, in a retrospective cohort study of 114 patients with cancer with a history of hepatitis B who received ICIs, 5.3% developed reactivation of hepatitis B virus.^76^ All six patients had undetectable viral DNA at baseline and received PD-1/PD-L1 blockade. Absence of antiviral prophylaxis was the only significant risk factor (OR 17.50, 95% CI 1.95 to 157.07, p=0.004). Notably, 12.7% were on concurrent corticosteroids, which were not a significant risk factor, suggesting ICI treatment may have been causative.^76^ Additionally, a systematic review of patients with hepatitis B or C revealed that, although ICIs were generally safe, 2.8% showed an increase viral load among 106 patients not on antiviral treatment.^78^ Furthermore, Lee *et al* retrospectively reviewed records of 62 patients with hepatitis B-related hepatocellular carcinoma. Of the six patients who did not receive antiviral treatment, one developed reactivation of hepatitis B after being treated with nivolumab, while none of the patients who had antiviral therapy developed reactivation of hepatitis B, suggesting this may prevent reactivation with ICI.^79^ Notably, viral titres do not appear to be monitored routinely in all centres. One review identified that only 6 out of 35 patients with hepatitis B/C infection had pretreatment and post-treatment viral titres available and did not identify any reactivation, though it is unclear if these patients were on antiviral therapy.^80^ Another retrospective analysis of 19 patients with a history of hepatitis B/C revealed only 7 patients had viral titre measurement after starting ICI treatment.^81^

While hepatitis B after ICI treatment can be associated with immunosuppression states,^74^ there is emerging evidence that excess inflammation can also promote viral replication. Immunological assays of chronic hepatitis B virus-infected patients have associated high concentrations of PD-1-expressing cytotoxic T lymphocytes with a reduction in acute flares of hepatitis B, while lower number of PD-1-expressing lymphocytes had a higher number of acute flares.^82^ Furthermore, PD-1-expressing lymphocytes were also shown to be functional and secrete IFN-γ, challenging an assumption that these cells are ‘exhausted’.^82^ Excess cytokine release by blocking PD-1/PD-L1 may result in destruction of hepatocytes, allowing escape of infectious, previously latent virus.^83^

Thus, PD-1-expressing lymphocytes appear to assist with viral control, while blockade of PD-1/PD-L1 signalling and the resulting hyperinflammatory state may disrupt balanced immune control established in latent hepatitis B infection and promote viral growth. While immunotherapy may be used safely in chronic hepatitis B infection, there is a subset of patients who may develop viral reactivation. Hence, appropriate screening and monitoring of hepatitis virus status is vital.

### Human Herpesviridae: CMV, varicella-zoster virus (VZV) and Epstein-Barr virus (EBV)

CMV, EBV and VZV are herpesviruses that establish a state of chronic latent infection in most humans by adulthood.^84^ While there are a number of reports of CMV following immunosuppression,^36–38^ reactivation of CMV,^85 86^ EBV^87^ and VZV^88 89^ in the absence of immunosuppression has also been reported, with features mimicking irAEs.^90^ In fact, some evidence suggests CMV may actually be an underlying trigger for severe irAEs, as CMV is found disproportionately among severe ICI-associated pneumonitis and colitis.^91 92^ A retrospective cohort study analysing checkpoint inhibitor pneumonitis (CIP) revealed that CMV pp65 positivity rate in patients with severe CIP was much higher than that in patients without or with mild ICI pneumonitis (91.7 vs 20%).^91^ A cohort study by Franklin *et al* revealed that CMV reactivation was present in all treatment refractory cases through detection of CMV DNA in biopsy or plasma, or CMV IgM.^92^

While immunosuppression could be the causative factor in some reported cases, the hyperinflammatory state can promote viral pathology. For example, in inflammatory bowel disease, colonic inflammation can impair natural killer cell function, and this, along with a damaged mucosa from immunopathology, can promote viral reactivation.^93^ This suggests a potential role for dysregulated inflammation in a subset of ICI-associated CMV infection. ICIs may boost virus-specific T-cell specific activity,^94^ and then inflammation driven by interleukin (IL)-6 and IL-17 may enhance viral persistence by protecting virus-infected cells from apoptosis through expression of Bcl-2 and Bcl-xL.^95 96^ Additionally, in macaques, CTLA-4/PD-1 blockade can cause reactivation of simian immunodeficiency virus (SIV).^97^ Overall, these reports argue for a low threshold for investigating for herpesvirus infection before starting immunosuppression for potential irAEs.

### Fungal infections

**Figure 4:**
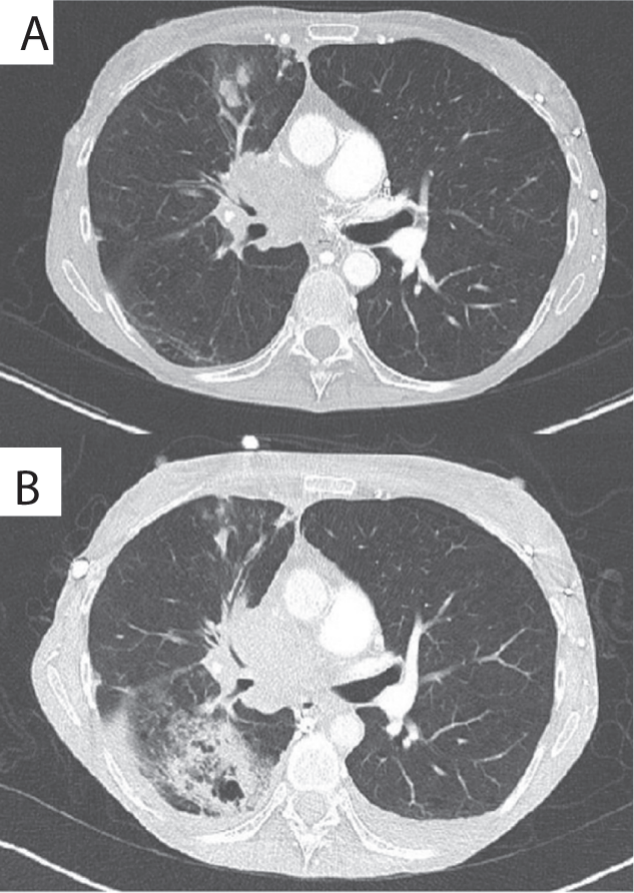
Development of pulmonary *Aspergillus* infection on nivolumab treatment. Original publication: adapted from Inthasot *et al*,^98^ reproduced with permission from the publisher.

While fungal infection can be a direct consequence of immunosuppression, an emerging number of cases of progressive fungal infection exacerbated by immunotherapy in the absence of immunosuppression are being reported, such as aspergillosis (figure 4).^98^ Dysregulated immunity appears to play a role, indicated by dramatic worsening of chronic progressive pulmonary aspergillosis and fulminant deterioration of nasal fungal sinusitis postimmunotherapy. Again, clinical presentation can mimic cancer progression, with pulmonary cavitation,^98 99^ erosive skull base lesions with bilateral cavernous sinus involvement^100^ and new pulmonary nodules with pleural effusion,^101^ and so cases may well be under-reported. In these situations, immune checkpoint blockade may cause an exaggerated immune response to fungal colonisation, which could promote fungal growth similar to recent studies in Mtb infection.^69^

### Clostridium difficile

Babacan and Tanvetyanon reported five cases of *Clostridium difficile* infection (CDI) and irAE colitis, and interestingly, four of the cases did not have prior exposure to antibiotics and the other case did not have any immunosuppressive treatment.^102^ Additionally, CDI preceding irAE colitis has been reported.^103 104^ Inflammatory bowel disease can present with CDI, even in the absence of immunosuppression,^105^ suggesting that excessive inflammation can favour CDI. Given the similar pathophysiology in IBD and irAE colitis,^106^ there may be a subset of patients that develop CDI because of the acquired hyperinflammatory state. Evolving evidence demonstrates that ICIs can alter the gut microbiota, and CDI may be a manifestation of this.^107^

### SARS-CoV-2

In addition to reactivating chronic infections, ICI therapy may worsen acute infections associated with immune asynchrony such as SARS-CoV-2. Patients with cancer may have higher risks of severe complication outcomes with SARS-CoV-2 infection regardless of ICI therapy.^108^ Interestingly, patients with cancer may also have a poor vaccine response, with reduced immunogenicity post a single dose of vaccine.^109^ Furthermore, patients with haematological malignancies in particular are at risk of reduced seroconversion, prolonged viral shedding and sustained immune dysregulation following SARS-CoV-2 infection.^110^ These factors make analysis challenging. However, some reports describe rapidly progressive SARS-CoV-2 following ICIs.^111 112^ In a retrospective analysis of 423 cases of patients with cancer diagnosed with SARS-CoV-2, ICI use was an independent risk factor for hospitalisation (univariate analysis OR 2.53, 5% CI 1.18 to 5.67, p=0.017; multivariate analysis OR 2.84, 95% CI 1.24 to 6.72, p=0.013) and requirement for high-flow oxygen support or mechanical ventilation (univariate analysis HR 2.38, 95% CI 1.29 to 4.38, p=0.005; multivariate analysis HR 2.38, 95% CI 1.29 to 4.38, p=0.004).^113^ The risk was different in lung cancer compared with other solid cancers. For lung cancer, 12/23 (52%) patients not on ICIs were hospitalised; 35% developed severe respiratory illness, compared with 10/12 (83%) of patients with lung cancer on ICIs, of whom 58% developed severe respiratory illness. Interestingly, only one of 31 ICI-treated patients had corticosteroid therapy prior to SARS-CoV-2 diagnosis. In contrast, systemic chemotherapy within 30 days was not a risk factor for hospitalisation or severity of illness in this study.^113^ Another Italian cohort study identified that ICI as well as chemotherapy increased the risk of hospitalisation and death due to SARS-CoV-2.^114^ Therefore, these series support an adverse interaction between ICIs and SARS-CoV-2 infection, although other studies have not replicated these findings.^115–118^ Additionally, it has been suggested that lung injury induced by SARS CoV-2 may increase the risk of subsequent irAE pneumonitis.^119^

In terms of the underlying mechanism, ICI-induced immune asynchrony is a potential explanation for more severe COVID-19 infection. Patients with SARS-CoV-2 infection who are admitted to the intensive care unit have higher plasma concentrations of IL-2, IL-6, IL-7, IL-10 and TNF-α, and severe SARS-CoV-2 is associated with a cytokine storm.^120–122^ Of note, high concentrations of inflammatory cytokines have predictive potential in irAEs and can also correlate with irAE severity in PD-1 immunotherapy.^123^ Additionally, cytokine release syndrome is a rare complication of PD-1 immunotherapy.^124^ Taken together, ICIs could promote a cytokine storm in SARS-CoV-2 via immune dysregulation, increasing the risk of a harmfully excessive response to the pathogen. Recent observations such as the potential benefits of tocilizumab, an anti IL-6 agent,^125^ support the hypothesis that severe COVID-19 infection is exacerbated by an asynchronous, dysregulated immune state. Clinically, it is important to distinguish between SARS-CoV-2 infection and irAE pneumonitis in patients on ICIs.^126^

## Conclusion

**Figure 5:**
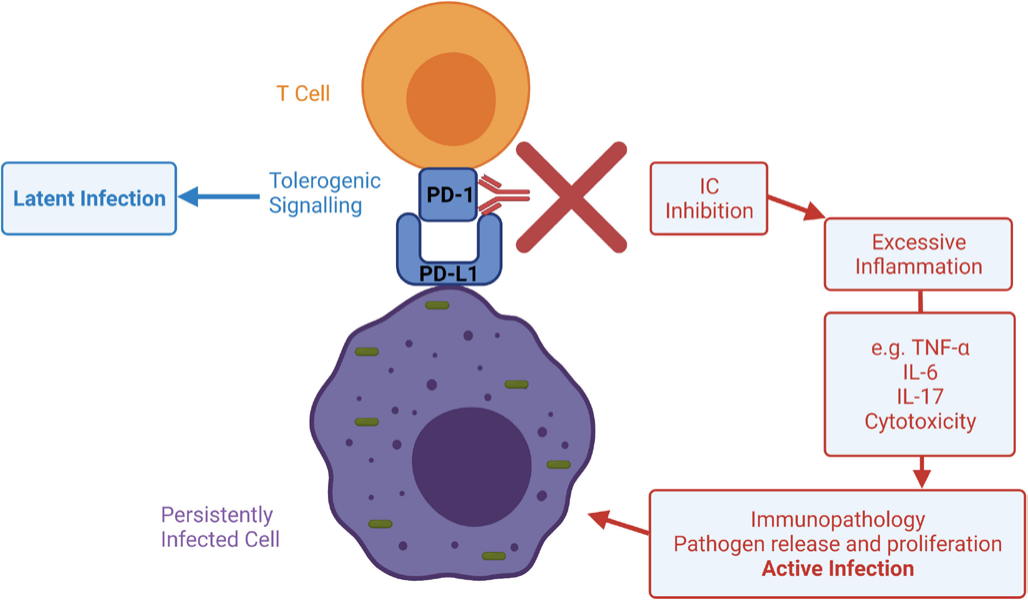
Potential mechanisms whereby ICIs may lead to reactivation of infection and immune-related tissue damage. Sustained antigen exposure from persistently infected cells can cause an overexuberant immune response harmful to the host. ICs may regulate homeostasis in latent infection, while signalling disruption with ICIs may promote excessive inflammation, infection reactivation and immunopathology. IC, immune checkpoint; ICI, immune checkpoint inhibitor; IL, interleukin; PD-1, programmed cell death protein 1.

ICIs are transformative drugs that improve survival in many forms of cancer, but a common side effect of immunotherapy is irAEs. These irAEs are treated with immunosuppression, which can cause opportunistic infection. However, an emerging paradigm is that ICIs can also lead to a new pattern of infections resulting from dysregulated immunity, which we term ITI-DI. Immune checkpoint activity may be necessary for the establishment of latent infection and a stable symbiosis between pathogen and host, and so neutralising these with ICIs may in fact increase infection-related immunopathology (figure 5).^127^ Screening for chronic infection such as latent TB and hepatitis should be considered before starting ICI therapy, and infection reactivation should be considered within the differential diagnosis even in the absence of immunosuppression.
