# Supplementary material for: Infections due to dysregulated immunity: an emerging complication of cancer immunotherapy
Source: Thorax. 2021 Oct 4;77(3):304–11. doi: 10.1136/thoraxjnl-2021-217260 (PMC8867274; doi:10.1136/thoraxjnl-2021-217260)
Supplement: Supplementary data [file thoraxjnl-2021-217260supp001.pdf]

Appendix 1 Prisma 2009 checklist summary

TITLE

Title: Systematic Review. ImmunoTherapy Infections due to Dysregulated Immunity (ITI-DI): A New Paradigm?

ABSTRACT

Abstract: Immune checkpoint inhibitors (ICIs) have revolutionised cancer treatment. However, immune related adverse events (irAEs) are a common side-effect, which can mimic infection. Additionally, treatment of irAEs with corticosteroids and other immunosuppressant agents can lead to opportunistic infection, which we have classed as ImmunoTherapy Infections due to ImmunoSuppression (ITI-IS). However, emerging reports demonstrate that some infections can be precipitated by ICIs in the absence of immunosuppressive treatment, in contrast to the majority of reported cases. These infections are characterised by a dysregulated inflammatory immune response, and so we propose they are described as ImmunoTherapy Infections due to Dysregulated Immunity (ITI-DI). This review summarises the rapidly emerging evidence of these phenomena and proposes a new framework for considering infection in the context of cancer immunotherapy.

INTRODUCTION

Rationale: Given that emerging evidence of increased infection in patients treated with ICIs, and evidence that immune checkpoint deficiency can be associated with recurrent infections, we were interested in evaluating the infectious sequelae of ICI therapy.

METHODS

Objectives: We undertook a systematic review following the Preferred Reporting Items for Systematic Reviews and Meta Analyses (PRISMA guidelines). We were interested primarily in reported cases of infection following immune checkpoint inhibitor initiation for cancer, to try and to understand any patterns of reported infections. We excluded any studies in children.

Protocol and registration: Full protocol in appendix 3. Registered on PROPSERO 8th March 2021 CRD 4202141634

Eligibility criteria: There are were no specific restrictions on the types of study design included though we anticipated the data would be mostly case series and cohort studies. The conditions of interest were infections occurring following cancer treatment with immune checkpoint inhibitors. Inclusion criteria: Reports of infection in adult cancer patients following immune checkpoint inhibitor, regardless of cancer type. Exclusion: Patients less than 18 years old. The intervention of interest is use of immune checkpoint inhibitor in treatment of cancer. Studies were eligible if they included documented cases of cancer patients who were found to have developed infections after taking immune checkpoint inhibitors.

Information sources: Medline Ovid 1996- February week 4 2021 to identify reports of infections post cancer immunotherapy initiation. We Also consulted Dr Gil Redelman-Sidi and Dr Kohei Fujita to identify any additional studies and also identified sources through references and citations.

Search: Medline (Ovid 1996- February week 4 2021), advanced search, keyword :("Infection" or "Infectious Disease") and ("Immune checkpoint inhibitor" or "PD-1" or "PD-L1" or "CTLA-4")

Study selection: Studies were eligible if they included documented cases of cancer patients who were found to have developed infections after taking immune checkpoint inhibitors. We obtained information on cancer type as well as immune checkpoint inhibitor used, and concurrent immunosuppression use, when available. Because the data being collected were expected to be largely case series and cohort studies, descriptive statistics were the primary tool of analysis. We compared the reported outcomes of various patients using the aforementioned data points to better assess for associations between the circumstances under which patients have developed infection while using immune checkpoint inhibitors.

Data collection process: TM performed the Medline Ovid search using the aforementioned search terms. Search results were merged using EndNote X9 (Clarivate Analytics) and deduplicated. TM screened the studies for inclusion using the Title, Abstract, Introduction and final paragraph, looking for evidence of cases of cancer patients who were found to have developed infections after taking immune checkpoint inhibitors. Full-text articles, for potential inclusion, were retrieved and tabulated by TM and independently double screened for eligibility by two authors (TM and PE) The final list of studies to be included was agreed by all four authors. In cases of uncertainty, the conflicts were resolved by TM and PE taking a conservative approach when deciding which studies to include.

Data items: Data were extracted by TM and verified by PE. The final data was discussed and agreed among all four authors. We extracted the following: authors, cancer type, immune checkpoint inhibitor used, immunosuppressive treatments used, characteristics of infection, number of patients. Data relating to immune related adverse events and descriptive statistics were also collected when available.

Risk of bias in individual studies: TM used Centre for Evidenced Based Medicine critical appraisal tools when reviewing studies for inclusion. The data collected were largely case series and cohort studies with reported individual outcomes though descriptive statistics were used when available as the primary level of analysis.

Synthesis of results : We undertook a narrative synthesis and themes were identified and discussed by TM and PE and agreed with all four authors. We compared the reported outcomes of various patients using the aforementioned data points to better assess for associations between the circumstances under which patients have developed infection while using immune checkpoint inhibitors.

Risk of bias across studies: There may be underreporting of cases as causality of infection with immune checkpoint inhibitors is not clearly established.

Additional analyses: Not applicable

RESULTS

Study selection: Prisma Diagram Figure 1

Study characteristics Table S1

Synthesis of results: Table 1, S1. We undertook a narrative synthesis and themes were identified We analysed reported outcomes using the aforementioned data points to compare the circumstances under which patients have developed infection while using immune checkpoint inhibitors Themes identified included Immunotherapy infections associated with Immunosuppression (ITI-IS) and Immunotherapy infections associated with dysregulated immunity (ITI-DI)

DISCUSSION

Summary of evidence: Presented in text page 6 -15

Limitations: There is likely underreporting of cases.

Conclusion: Presented in text. Pages 15

Appendix 2 Review Protocol

Review question

Is there any association between use of immune checkpoint inhibitors in cancer patients and development of infections? If so are there different patterns of infections?

Searches

Medline (Ovid 1996- February week 4) was searched on 8th March 2021

Types of study to be included

There are no restrictions on the types of study design that we will include.

Condition or domain being studied

The conditions of interest are infections occurring following cancer treatment with immune checkpoint inhibitors.

Participants/population

Inclusion: Adult cancer patients are the subject of this review, regardless of cancer type.

Exclusion: Patients less than 18 years old.

Intervention(s), exposure(s)

The intervention of interest is use of immune checkpoint inhibitor in treatment of cancer.

Comparator(s)/control

Not applicable.

Main outcome(s)

Development of Infection

**** Measures of effect***

Not applicable

Additional outcome(s)

Not applicable

**** Measures of effect***

Not applicable

Data extraction (selection and coding)

Studies will be eligible if they include documented cases of cancer patients who were found to have developed infections after taking immune checkpoint inhibitors. We will obtain information on cancer type as well as immune checkpoint inhibitor used, immunosuppressive treatments use.

Risk of bias (quality) assessment

Our primary method of assessing the quality of the studies will involve the level of detail relayed in the descriptions of the cases of infection. In assessing these cases, we will be using Centre for Evidenced Based Medicine critical appraisal tools. Dr. Morelli and Professor Elkington will be involved in quality assessment and will take a conservative approach when deciding what to include in the event of any disagreements about a study's quality and eligibility for inclusion.

Strategy for data synthesis

Because the data being collected are expected to be largely case series and cohort studies, descriptive statistics will be the primary tool of analysis. We will be comparing the reported outcomes of various patients using the aforementioned data points to better assess for associations between the circumstances under which patients have developed infection while using immune checkpoint inhibitors.

Analysis of subgroups or subsets

We will investigate whether there are any differences in outcomes noted on the basis of cancer type, immune checkpoint inhibitor used, and how the infection developed, including if there was any concurrent immunosuppressive treatment.

Contact details for further information

T.G.Morelli@soton.ac.uk

Organisational affiliation of the review

University Hospital Southampton

Review team members and their organisational affiliations

Dr Tommaso Morelli

Professor Paul Elkington

Type and method of review

Systematic review

Funding sources/sponsors

NIHR

MRC

Conflicts of interest

Language

English

Country

United Kingdom

Table S1: Clinical studies of Immunotherapy associated infections

Author	Type of Study	Cancer	ICI	irAEs	Immunosuppression	No of Patients/percentage	Significant Findings	Infection
(Lord, Hackman et al. 2010)	Case Series	Prostate	Ipilimumab	Colitis	Corticosteroids, Infliximab, tacrolimus, rapamycin	1	Refractory irAE colitis immunosuppression resulted in disseminated *Aspergillus fumigatus* infection	*Aspergillus fumigatus*
(Kyi, Hellmann et al. 2014)	Case Report	Melanoma	Ipilimumab	Colitis	Corticosteroids, Infliximab	1	Fatal invasive pulmonary aspergillus	*Aspergillus fumigatus*
(Arriola, Wheater et al. 2015)	Case Report	Melanoma	Ipilimumab	Colitis	Corticosteroids, Infliximab 1 patient had stable CLL	2	Pneumocystis jirovecii Pneumonia recovery with treatment	Pneumocystis jirovecii (2) *Cytomegalovirus(1)*
(Uslu, Agaimy et al. 2015)	Case Report	Melanoma	Ipilimumab	Colitis	Corticosteroids, Infliximab	1	*Cytomegalovirus* hepatitis recovered with treatment	*Cytomegalovirus*
(Gupta and Khanna 2015)	Case Report	Non -Hodgkin Lymphoma	Combined Ipilimumab +Nivolumab	Colitis following C*lostridium difficile* infection	Corticosteroids	1	Clostridium difficile preceeded severe irAE colitis	*Clostridium difficile*
(Rizvi, Mazieres et al. 2015)	Trial	NSCLC	Nivolumab	Pneumonitis	Not stated	2	Pneumonia and VZV infection	Pneumonia *Varicella Zoster*
(Robert, Long et al. 2015)	Trial	Melanoma	Nivolumab	Not stated	Not stated	8	-	8 cases of drug related infection reported
(Rosenberg, Hoffman-Censits et al. 2016)	Trial	Bladder cancer	Atezolizumab	Not stated	Not stated	1	-	1 case of sepsis

(Del Castillo, Romero et al. 2016)	Cohort	Melanoma	Ipilimumab (73%), Nivolumab 5.7%), Pembrolizumab (9.2%), Combined ipilimumab +Nivolumab (8.9%)	Not stated	Corticosteroids, Infliximab (both statistically significant risk)	54 infections from 740 patients (7%)	Risk Factors: corticosteroids (odds ratio [OR], 7.71, 3.71–16.18; P < 0.0001)<br><br>Infliximab (OR, 4.74; 2.27–9.45; P < 0.0001)<br><br>Age, sex, and prior receipt of chemotherapy not risk factors	Bacterial pneumonia (13), bacteraemic sepsis (13) *Clostridium difficile (10), other bacterial (10) Aspergillus fumigatus,(2) Pneumocystis jirovecii (3),Candida albicans (1), Varicella zoster (3), cytomegalovirus (1), Epstein Barr Virus, (1) Strongyloides(1)*
(Lee, Chan et al. 2016)	Case Report	Hodgkin Lymphoma	Nivolumab	None	Lymphoma	1	Pulmonary TB	*Mycobacterium tuberculosis*
(Herbst, Baas et al. 2016)	Trial	NSCLC	Pembrolizumab	None	None	complications of pneumonia (1.5%), lung infection (0.3%), oral candidiasis (0.3%) and urinary tract infection (0.3%).	-	*Pneumonia Candida UTI*
(Fujita, Terashima et al. 2016)	Case Report	NSCLC	Nivolumab	None	None	1	Pulmonary TB	*Mycobacterium tuberculosis*
(Lankes, Hundorfean et al. 2016)	Case Report	Melanoma	Ipilimumab + Nivolumab	Colitis	Cortiosteroid, Infliximab	1	CMV colitis	*Cytomegalovirus*

(Uchida, Fujita et al. 2017)	Case Report	NSCLC	Nivolumab	1	Acute progression of chronic progressive pulmonary aspergillosis	1	Acute progression of chronic progressive pulmonary aspergillosis	*Aspergillus fumigatus*
(Fuentes and Al-ahwel 2017)	Case Report	NSCLC	Nivolumab	None	None	1	Pulmonary Mycobacterium avium intracellulare	*Mycobacterium intracellulare*
(Chu, Fang et al. 2017)	Case Report	NSCLC	Nivolumab			1	Pericardial Mtb	*Mycobacterium tuberculosis*
(Koksal, Toka et al. 2017)	Case Report	Melanoma	Ipilimumab+Nivolumab	None	None	1	Hepatitis B reactivation	*Hepatitis B*
(Ragunathan, Dadana et al. 2017)	Case Report	NSCLC	Pembrolizumab	Hepatitis	Corticosteroid	1	Hepatitis B	*Hepatitis B*
(Lake 2017)	Case Report	NSCLC	Nivolumab	None	HIV	1	Hepatitis B	*Hepatitis B*
(Franklin, Rooms et al. 2017)	Cohort	Melanoma	Ipilimumab (4 patients), Dual Ipilimumab and Nivolumab (1 patient)	Colitis	Corticosteroids, infliximab, ciclosporin	5 (12.2%)	CMV Colitis	*Cytomegalovirus*
(Lu, Firpi-Morell et al. 2018)	Case Report	Bladder Cancer	Atezolizumab, followed by Pembrolizumab	None	None	1	CMV gastritis	*Cytomegalovirus*
(Redelman-Sidi, Michielin et al. 2018)	Consensus Document Review	N/A	PD-1/L1<br>CTLA-4	N/A	Corticosteroids and TNF-α blockers cited as cause for opportunistic ICI infection	N/A	Consensus ICIs do not independently increase infection risk after examining Del Castillo cohort and RCT data, suggested screening for Mtb, Hepatitis B/C prior to ICI initiation, recommended PCP prophylaxis for irAE treatment	Opportunistic Infections due to immunosuppression
(Martinot, Ahle et al. 2018)	Case Report	Hodgkin's Lymphoma	Nivolumab	None	Corticosteroid<br>Lymphoma	1 in case report	Severe Progressive multifocal leukoencephalopathy due to *JC polyoma virus*<br>Case report highlights 4 other	Opportunistic<br>*JC polyoma virus*

							unpublished cases in WHO/ EudraVigilance registry data	
(Picchi, Mateus et al. 2018)	Case Series	1 NSCLC, 1 Melanoma	1 Nivolumab, 1 Pembrolizumab	None	None	2	1 pleural TB<br>1 spinal TB	*Mycobacterium tuberculosis*
(He, Zhang et al. 2018)	Case Report	Melanoma	Pembrolizumab	None	None	1	Pulmonary TB	*Mycobacterium tuberculosis*
(Jensen, Persson et al. 2018)	Case Report	NSCLC	Nivolumab	None	None	1	Pulmonary TB	*Mycobacterium tuberculosis*
(Elkington, Bateman et al. 2018)	Case Report	Melanoma	Pembrolizumab	None	None	1	Pulmonary and hepatic TB	*Mycobacterium tuberculosis*
(Tetikkurt, Taş et al. 2018)	Case Report	Oral SCC	Pembrolizumab	None	Not stated	1	Pulmonary TB	*Mycobacterium tuberculosis*
(Pandey, Ezemenari et al. 2018)	Case Report	NSCLC	Pembrolizumab	Hepatitis	Corticosteroid	1	Hepatitis B reactivation	*Hepatitis B virus*
(Fujita, Kim et al. 2019)	Cohort	NSCLC	Nivolumab	Not Stated	Corticosteroids, previous chemotherapy not statically significant risk factors		33 infections in 32/167 patients (19.2%)<br><br>78.1% bacterial (*Streptococcus pneumoniae, Haemophilus influenzae, Klebsiella pneumoniae,* methicillin resistant *Staphylococcus aureus (MRSA),* methicillin sensitive *Staphylococcus aureus (MSSA) Staphylococcus schleiferi*, *Mycobacterium tuberculosis* and other unknown presumed bacterial infection)<br>6.3% fungal (*Aspergillus*	Bacterial pneumonia (17), Mycobacterium tuberculosis (1), lung abscess (2),bacteriaemic sepsis (2), Aspergillus fumigatus (1), Candida Albicans (1), Varicella zoster virus (2), *Influenzae* (4)

							fumigatus and candida)<br>18.8% viral(*Varicella zoster, Influenzae).*<br><br>Diabetes mellitus significant risk factor (OR 3.61 1.14-11.4 p= 0.028)	
(Zhang, Zhou et al. 2019)	Cohort	Nasopharyngeal carcinoma (35, 24.6%), hepatocellular carcinoma (28, 24.6%), Melanoma (14, 12.3%), NSCLC; *n* = 13, 11.4%)	Anti-PD-1/PD-L1 72.8%, whereas. 27.2% combined PD-1, CTLA-4	Not stated	14 (corticosteroid, not significant risk factor	Six patients (5.3%) developed HBV reactivation with PD-1/L1 blockade. lack of antiviral prophylaxis was significant risk factor reactivation OR 17 .5	Hepatitis B	*Hepatitis B virus*
(Gupta, Tun et al. 2019)	Case Report	NSCLC	Durvalumab	None	Corticosteroids	1	Pleural Aspergillus fumigatus improved with treatment.<br><br>One month prior had 5 day course only of prednisolone 50mg for COPD exacerbation	*Aspergillus fumigatus*
(Oltolini, Ripa et al. 2019)	Case Report	NSCLC	Pembrolizumab	None	Corticosteroids	1	Fatal Invasive pulmonary Aspergillosis, *Stenotrophomonas maltophilia* pneumonia, *Pseudomonas*	Opportunistic *Aspergillus fumigatus, Stenotrophomonas maltophilia, Pseudomonas aeruginosa, Cytomegalovirus*

							aeruginosa pneumonia, *cytomegalovirus* pneumonitis<br><br>Following dexamethasone for metastases	
(Schwarz, Kocher et al. 2019)	Case Series	NSCLC	Nivolumab	Pneumonitis	Corticosteroids, Mycophenolate moferil	2	2 fatal *Pneumocystis, 1 cytomegalovirus jirovecii* pneumonia following immunosuppression for suspected irAE pneumonitis	Opportunistic Pneumocystis jirovecii (2) *Cytomegalovirus(1)*
(Babacan and Tanvetyanon 2019)	Case Series	NSCLC	Tremelumab, durvalumab, Nivolumab	Colitis	Corticosteroid, Adalimumab, Infliximab<br>(1 pt no immunosuppression)	5	4 patients developed clostridium difficile following immunosuppression irAE colitis without any antibiotics, 1 patient developed Clostridium difficile without any immunosuppression	*Clostridium difficile*
(Zhou, Klionsky et al. 2019)	Case Report	NSCLC	Pembrolizumab	Colitis following C*lostridium difficile* infection	Corticosteroids	1	Clostridium difficile preceeded severe irAE colitis	*Clostridium difficile*
(Ferguson, Heberton et al. 2019)	Case Report	Melanoma	Pembrolizumab	None	None	1	Disseminated Blastomycosis	Blastomycosis
(van Eeden, Rapoport et al. 2019)	Case Report	NSCLC	Nivolumab	None	None	1	Pulmonary TB	*Mycobacterium tuberculosis*
(Barber, Sakai et al. 2019)	Case Report	1 Merkel Cell Ca, 1 Nasopharyngeal Ca	Pembrolizumab, Nivolumab (PD-10	None	None	2	Mtb	*Mycobacterium tuberculosis*
(Takata, Koh et al. 2019)	Case Report	NSCLC	Nivolumab	None	None	1	Mtb	*Mycobacterium tuberculosis*
(Gueguen, Bailly et al. 2019)	Case Report	Melanoma	Pembrolizumab	Colitis	Corticosteroid<br>Mycophenolate<br>Cyclosporine	1	CMV colitis	*Cytomegalovirus*

(Sakoh, Kanzaki et al. 2019)	Case Report	NSCLC	Nivolumab	none	Developed VZV otitis media prior to prednisolone	1	VZV otitis media and cerebral VZV	*Varicella zoster virus*
(Assi, Danu et al. 2019)	Case Report	Lymphoma	Pembrolizumab	None	None	1	VZV shingles	*Varicella zoster virus*
(Saikawa, Nagashima et al. 2019)	Case Report	SCLC	Pembrolizumab	None	None prior to EBV diagnosis	1	EBV induced cerebellar ataxia	*Epstein-Barr virus*
(Shah, Al-Shbool et al. 2019)	Cohort	Various	PD-1	Various	Not stated	16 HIV, 29 HBV/HCV	Only 6 patients pre and post ICI viral load recorded, no viral reactivation recorded, unclear antiviral status	*Hepatitis B Virus*
(Fujiwara, Kuchiba et al. 2020)	Review of Phase I Trial data	Various	Not stated	Not Stated	Not Stated	18/100 patients had infectious adverse event (18%), 5 grade 3 or above, Odds ratio of infection related adverse event similar to those of molecular targeted agents for cancer	Not Stated	Not Stated
(Karam, Noel et al. 2020)	Registry	Melanoma NSCLC	PD-1/L1	Not stated	Corticosteroids not significant risk factor	200	18% had post immunotherapy infection	Pulmonary infection<br>Skin infection<br>UTI<br>GI infection
(Kanjanapan and Yip 2020)	Cohort	Melnaoma NSCLC	PD-L1<br>CTLA4<br>Combination	Not stated	Corticosteroids not a significant risk factor	27% patients had an infection up to one year post ICI	Cutaneous 24%<br>Genitourinary 33%<br>Respiratory 29%<br>Bacteraemia 9%<br>Gastrointestinal 4%	Various bacterial, viral and fungal identified through culture/PCR
(Lee, Chao et al. 2020)	Cohort	Hepatocellular Carcinoma	Nivolumab	Not stated	Not stated	1 patient reactivation of hep B	Notably no patients on antivirals had hep b reactivation	Hepatitis B Virus
(Anand, Sahu et al. 2020)	FAERS Registry	Mtb Cases<br>Lung (44, 61.11%), Head and Neck (6, 8.33%), Gastric (6, 8.33%), Hodgkins Lymphoma (3, 4.16%), Melanoma (1, 2.7%,)	Mtb Cases<br>Nivolumab (45), Pembrolizumab (18), Atezolizumab (5), Durvalumab (4).<br><br>AMI Cases<br>Nivolumab (9), pembrolizumab (2), atezolizumab (1), du rvalumab (1)	Not stated	Not stated	72 TB<br>ROR of TB with PD-1/PD-L1 inhibitors was 1.79<br><br>13 atypical mycobacterial infection (AMI), ROR of AMI infection with PD-1/PD-L1 inhibitors was 5.49	Mtb, AMI	*Mycobacterium tuberculosis*<br>Atypical mycobacterial infection

		Pancreatic (1, 2.7%), Ovarian (1, 2.7%) , Neuroendocrine (1, 2.7%), Myeloma (1, 2.7%), Renal (1, 2.7%), Transitional Cell (1, 2.7%) Unknown (1, 2.7%)<br><br>AMI Cases<br>Lung 10 76.2%, Head and Neck 1 7.69%, Melanoma 1 7.69%, Unknown 1 7.69%						
(Fujita, Yamamoto et al. 2020)	Cohort	NSCLC	Pembrolizumab, Nivolumab , Durvalumab	Not Stated	Not Stated	5/197 patients 1.7% developed active TB	(60%) pulmonary Mtb and 2 (40%) extrapulmonary Mtb (cervical and hilar lymph node, knee arthritis).	*Mycobacterium tuberculosis*
(Dai, Liu et al. 2020)	Cohort	Lung Cancer<br>GI Cancer<br>Breast Cancer, Thyroid Cancer,	PD-1 inhibitors not specified	Not stated	Not stated	6 patients on ICI	2/6 patients died<br>4/6 critical symptoms	*SARS Co-V-2*
(Im, Lee et al. 2020)	Cohort	NSCLC	Pembrolizumab, Nivolumab (PD-1), Atezolizumab (PD-L1)	2 irAE thyroiditis	Corticosteroids (2 patients)	3	Mtb incidence rate 394.4 (compared to 51.5 in local population)	*Mycobacterium tuberculosis*
(Chan, Gwee et al. 2020)	Cohort	NSCLC	Durvalumab (PD-L1), Pembrolizumab	1 patient who acquired Mtb following ICI had irAE arthritis	Not stated	4 patients	4/191 (2.09%) of patients developed Mtb reactivation	*Mycobacterium tuberculosis*
(Pertejo-Fernandez, Ricciuti et al. 2020)	Cohort	NSCLC	PD-1/L1	6 patients irAE hepatitis	Not stated	19 patients, 3 with chronic hepatitis B all on antiviral therapy	No hepatitis B reactivation in this study	*Hepatitis B Virus*

(Liu, Liu et al. 2020)	Case Series	NSCLC	Nivolumab (1),<br>Pembrolizumab (2)<br>Toripalimab (1)	Pneumonitis	Corticosteroids	4	1 case of combined pulmonary *Pneumocystis jirovecii, Aspergillus fumigatus* and *cytomegalovirus* following nivolumab, improved with treatment<br><br>1 case of *Corynebacterium striatum* and *Candida albicans* following corticosteroid for irAE pneumonitis following pembrolizumab improved with treatment<br><br>1 case of fatal Pneumocystis jirovecci pneumonia, cytomegalovirus following corticosteroid for suspected radiation pneumonitis<br><br>1 case of combined *Pneumocystis jirovecii, Pseudomonas aeruginosa* and *Candida albicans* following Toripalimab and corticosteroid for suspected irAE pneumonitis	Opportunistic *Pneumocystis jirovecii(3)i, Aspergillus fumigatus Candida albicans(2) Corynebacterium striatum(1) Pseudomonas aeruginosa(1) Cytomegalovirus(1)*
(Lee, Shaw et al. 2020)	Case Series	Not Stated	Not Stated	Colitis	Specific Immunosuppression not stated	5	5 patients Campylobacteriosis<br>1 no immunosuppression	*Campylobacter*

							but had received antibiotics<br>4 following unspecified irAE treatment for irAE colitis	
(Krane, Beswick et al. 2020)	Case Report	Melanoma	Pembrolizumab	None	None	1	non-invasive allergic fungal sinus disease.	*Aspergillus fumigatus*
(Inthasot, Bruyneel et al. 2020)	Case Report	NSCLC	Pembrolizumab<br>Nivolumab	None	None	2	1 invasive pulmonary aspergillosis<br>1 pulmonary Mycobacterium tuberculosis	*1 Aspergillus fumigatus*<br>*1 Mycobacterium tuberculosis*
(Si, Erickson et al. 2020)	Case Report	Non-Hodgkin Lymphoma	Pembrolizumab	None	Post autologous stem cell transplant	1	*Pneumocystis jirovecii* pneumonia recovered with treatment	Opportunistic *Pneumocystis jirovecii*
(Malek, Taremi et al. 2020)	Case Report	Renal Cell Carcinoma	Ipilimumab and Nivolumab	Hepatitis	Corticosteroids,<br>Rituximab,<br>Mycophenolate Mofetil	1	Severe invasive soft tissue aspergillosis following immunosuppression for refractory irAE hepatitis	Opportunistic *Aspergillus fumigatus*
(Taima, Tanaka et al. 2020)	Case Report	NSCLC	Durvalumab	Pneumonitis	Corticosteroids	1	Severe invasive pulmonary aspergillosis following corticosteroid for suspected irAE pneumonitis	Opportunistic *Aspergillus fumigatus*
(Fujita, Yamamoto et al. 2020)	Case Report	NSCLC	Nivolumab<br>Atezolizumab	None	None	3	3 cases of pulmonary MAI	*Mycobacterium intracellulare*
(Crawley, Breen et al. 2020)	*Case Report*	*NSCLC*	*Pembrolizumab*	*None*	*Corticosteroid*<br>*Carboplatin*<br>*Pemetrexed*	*1*	Pulmonary TB	*Mycobacterium tuberculosis*
(Murakami, Usui et al. 2020)	Case Report	NSCLC	Pembrolizumab	None	None	1	Pulmonary TB	*Mycobacterium tuberculosis*
(Suliman, Bek et al. 2020)	Case Report	NSCLC	Pembrolizumab	None	None	1	Pulmonary TB	*Mycobacterium tuberculosis*

(Pu, Yin et al. 2020)	Systematic Review	Hepatocellular carcinoma (124, 66.7%), Melanoma (46,24.7%), NSCLC (7, 3.8%)	137 PD-1 inhibitor monotherapy (nivolumab or pembrolizumab), 35 CTLA-4 monotherapy (Ipilimumab or tremelimumab). 1 Atezolizumab 5 (2.7%) received anti-PD-1, and anti-CTLA-4 combination therapy and the rest 4 (2.2%)	186 side effects reported. Unclear how many irAEs	Not stated	3 patients<br>2.8% patients not on antivirals increased viral load	Hepatitis B reactivation	*Hepatitis B Virus*
(Godbert, Petitpain et al. 2020)	Case Report	NSCLC	Durvalumab	None	None	1	Hepatitis B reactivation	Rapidly progressive and fatal
(Furuta, Miyamoto et al. 2020)	Case Report	Melanoma	Ipilimumab	Colitis	Corticosteroid	1	CMV colitis	*Cytomegalovirus*
(Kim, Ha et al. 2020)	Case Report	Melanoma	Pembrolizumab	None	None	1	CMV gastritis	*Cytomegalovirus*
(Robilotti, Babady et al. 2020)	Cohort	Various	Various	None	None	423	SARS-Co-V-2 higher hospitalisation and ICU admission	*SARS-Co-V-2*
(Zhai and Zhang 2020)	Case Report	Nasopharyngeal SCC	Sintilimab	None	None	1	SARS-Co-V-2	*SARS-Co-V-2*
(Bonomi, Ghilardi et al. 2020)	Case Report	NSCLC	Nivolumab	None	None	1	SARS-Co-V-2	*SARS-Co-V-2*
(Szabados, Abu-Ghanem et al. 2020)	Case Series	Urothelial cancer<br>Renal cancer	Atezolizumab<br>Ipilimumab and nivolumab	2 patients irAE pneumonitis and treated with corticosteroids	Corticosteroids	4	4 cases of mild covid	*SARS-Co-V-2*
(Pala, Conforti et al. 2021)	Case Report	Melanoma	Pembrolizumab	None	None	1	1 case of mild covid	*SARS-Co-V-2*
(Burns, Muhsen et al. 2021)	Registry	Various	Pembrolizumab	Not stated	Not stated	ROR of Hepatitis B reactivation with Pembrolizumab 2.32 (95% CI: 1.11-4.28) (P=0.013)	Hepatitis B reactivation	*Hepatitis B Virus*
(Lin, Lu et al. 2021)	Cohort	NSLCC	Pembrolizumab, Nivolumab, Topiralumab	12 pneumonitis	Corticosteroid	11	positivity rate of CMV pp65 in severe ICI pneumonitis patients was higher than that in no or	*Cytomegalovirus*

							mild CIP patients (91.7 vs 20%) (*P* < 0.01)	
(Sirgiovanni, Hinterleitner et al. 2021)	Case Report	Small Cell lung cancer	Nivolumab and Ipilimumab	None	None	1	Pulmonary TB	*Mycobacterium tuberculosis*
(Dipasquale, Persico et al. 2021)	Case Report	Squamous head and Neck Cancer	Anti PD-L1, not specified	irAE pneumonitis	None	1	Authors suggest lung injury induced by SARS-Co-V-2 increased risk of subsequent irAE pneumonitis	SARS-Co-V-2

Anand, K., G. Sahu, E. Burns, A. Ensor, J. Ensor, S. R. Pingali, V. Subbiah and S. P. Iyer (2020). "Mycobacterial infections due to PD-1 and PD-L1 checkpoint inhibitors." ESMO Open **5**(4).

Arriola, E., M. Wheater, R. Krishnan, J. Smart, V. Foria and C. Ottensmeier (2015). "Immunosuppression for ipilimumab-related toxicity can cause pneumocystis pneumonia but spare antitumor immune control." OncoImmunology **4**(10): e1040218.

Assi, T., A. Danu, C. Mateus, C. Robert, J.-M. Michot, T. Ibrahim, J. Lazarovici, D. Ghez, J. Rossignol, P. Dartigues, M. Terroir-Cassou-Mounat and V. Ribrag (2019). "Post-shingles granulomatous dermatosis related to anti-programmed cell death 1." Immunotherapy **11**(7): 591-598.

Babacan, N. A. and T. Tanvetyanon (2019). "Superimposed Clostridium difficile Infection during Checkpoint Inhibitor Immunotherapy-induced Colitis." Journal of Immunotherapy **42**(9): 350-353.

Barber, D. L., S. Sakai, R. R. Kudchadkar, S. P. Fling, T. A. Day, J. A. Vergara, D. Ashkin, J. H. Cheng, L. M. Lundgren, V. N. Raabe, C. S. Kraft, J. J. Nieva, M. A. Cheever, P. T. Nghiem and E. Sharon (2019). "Tuberculosis following PD-1 blockade for cancer immunotherapy." Sci Transl Med **11**(475).

Bonomi, L., L. Ghilardi, E. Arnoldi, C. A. Tondini and A. C. Bettini (2020). "A Rapid Fatal Evolution of Coronavirus Disease-19 in a Patient With Advanced Lung Cancer With a Long-Time Response to Nivolumab." Journal of thoracic oncology : official publication of the International Association for the Study of Lung Cancer **15**(6): e83-e85.

Burns, E. A., I. N. Muhsen, K. Anand, J. Xu, G. Umoru, A. N. Arain and M. Abdelrahim (2021). "Hepatitis B Virus Reactivation in Cancer Patients Treated With Immune Checkpoint Inhibitors." J Immunother.

Chan, G. H., Y. X. Gwee, J. L. Low, Y. Huang, Z. Y. Chan, J. R. Choo, N. Y. Ngoi, L. E. A. Y, V. Muthu, W. Q. Chong, A. Wong and R. A. Soo (2020). "Immune checkpoint inhibition for non-small cell lung cancer in patients with pulmonary tuberculosis or Hepatitis B: Experience from a single Asian centre." Lung Cancer **146**: 145-153.

Chu, Y. C., K. C. Fang, H. C. Chen, Y. C. Yeh, C. E. Tseng, T. Y. Chou and C. L. Lai (2017). "Pericardial Tamponade Caused by a Hypersensitivity Response to Tuberculosis Reactivation after Anti-PD-1 Treatment in a Patient with Advanced Pulmonary Adenocarcinoma." J Thorac Oncol **12**(8): e111-e114.

Crawley, D., R. A. Breen, P. T. Elkington and E. Karapanagiotou (2020). "Tuberculosis associated with Triplet therapy for lung cancer." Thorax **75**(7): 609-610.

Dai, M., D. Liu, M. Liu, F. Zhou, G. Li, Z. Chen, Z. Zhang, H. You, M. Wu, Q. Zheng, Y. Xiong, H. Xiong, C. Wang, C. Chen, F. Xiong, Y. Zhang, Y. Peng, S. Ge, B. Zhen, T. Yu, L. Wang, H. Wang, Y. Liu, Y. Chen, J. Mei, X. Gao, Z. Li, L. Gan, C. He, Z. Li, Y. Shi, Y. Qi, J. Yang, D. G. Tenen, L. Chai, L. A. Mucci, M. Santillana and H. Cai (2020). "Patients with Cancer Appear More Vulnerable to SARS-CoV-2: A Multicenter Study during the COVID-19 Outbreak." Cancer discovery **10**(6): 783-791.

Del Castillo, M., F. A. Romero, E. Argüello, C. Kyi, M. A. Postow and G. Redelman-Sidi (2016). "The Spectrum of Serious Infections Among Patients Receiving Immune Checkpoint Blockade for the Treatment of Melanoma." Clinical infectious diseases : an official publication of the Infectious Diseases Society of America **63**(11): 1490-1493.

Dipasquale, A., P. Persico, E. Lorenzi, D. Rahal, A. Santoro and M. Simonelli (2021). "COVID-19 lung injury as a primer for immune checkpoint inhibitors (ICIs)-related pneumonia in a patient affected by squamous head and neck carcinoma treated with PD-L1 blockade: a case report." J Immunother Cancer **9**(2).

Elkington, P. T., A. C. Bateman, G. J. Thomas and C. H. Ottensmeier (2018). "Implications of Tuberculosis Reactivation after Immune Checkpoint Inhibition." American Journal of Respiratory and Critical Care Medicine **198**(11): 1451-1453.

Ferguson, I., M. Heberton, L. Compton, J. Keller and L. Cornelius (2019). "Disseminated blastomycosis in a patient on pembrolizumab for metastatic melanoma." JAAD case reports **5**(7): 580-581.

Franklin, C., I. Rooms, M. Fiedler, H. Reis, L. Milsch, S. Herz, E. Livingstone, L. Zimmer, K. W. Schmid, U. Dittmer, D. Schadendorf and B. Schilling (2017). "Cytomegalovirus reactivation in patients with refractory checkpoint inhibitor-induced colitis." European Journal of Cancer **86**: 248-256.

Fuentes, F. and Y. Al-ahwel (2017). "Emerging Side Effects of Programmed Cell Death 1 Ligand Inhibitors: MAC Infection and Nivolumab." Chest **152**(4, Supplement): A678.

Fujita, K., Y. H. Kim, O. Kanai, H. Yoshida, T. Mio and T. Hirai (2019). "Emerging concerns of infectious diseases in lung cancer patients receiving immune checkpoint inhibitor therapy." Respir Med **146**: 66-70.

Fujita, K., T. Terashima and T. Mio (2016). "Anti-PD1 Antibody Treatment and the Development of Acute Pulmonary Tuberculosis." Journal of Thoracic Oncology: Official Publication of the International Association for the Study of Lung Cancer **11**(12): 2238-2240.

Fujita, K., Y. Yamamoto, O. Kanai, M. Okamura, M. Hashimoto, K. Nakatani, S. Sawai and T. Mio (2020). "Incidence of Active Tuberculosis in Lung Cancer Patients Receiving Immune Checkpoint Inhibitors." Open Forum Infect Dis **7**(5): ofaa126.

Fujita, K., Y. Yamamoto, O. Kanai, M. Okamura, K. Nakatani and T. Mio (2020). "Development of Mycobacterium avium Complex Lung Disease in Patients With Lung Cancer on Immune Checkpoint Inhibitors." Open forum infectious diseases **7**(3): ofaa067-ofaa067.

Fujiwara, Y., A. Kuchiba, T. Koyama, R. MacHida, A. Shimomura, S. Kitano, T. Shimizu and N. Yamamoto (2020). "Infection risk with PI3K-AKT-mTOR pathway inhibitors and immune checkpoint inhibitors in patients with advanced solid tumours in phase i clinical trials." ESMO Open **5**(2).

Furuta, Y., H. Miyamoto, H. Naoe, M. Shimoda, Y. Hinokuma, T. Miyamura, A. Miyashita, S. Fukushima, M. Tanaka and Y. Sasaki (2020). "Cytomegalovirus enterocolitis in a patient with refractory immune-related colitis." Case Reports in Gastroenterology **14**(1): 103-109.

Godbert, B., N. Petitpain, A. Lopez, Y. E. Nisse and P. Gillet (2020). "Hepatitis B reactivation and immune check point inhibitors." Dig Liver Dis.

Gueguen, J., E. Bailly, L. Machet, E. Miquelestorena-Standley, K. Stefic, P. Gatault and M. Büchler (2019). "CMV disease and colitis in a kidney transplanted patient under pembrolizumab." European Journal of Cancer **109**: 172-174.

Gupta, A. and S. Khanna (2015). "Ipilimumab-associated colitis or refractory Clostridium difficile infection?" BMJ Case Rep **2015**.
Gupta, A., A. Tun, K. Ticona, A. Baqui and E. Guevara (2019). "Invasive aspergillosis in a patient with stage III (or 3a or 3b) non-small-cell lung cancer treated with durvalumab." Case Reports in Oncological Medicine **2019 (no pagination)**.
He, W., X. Zhang, W. Li, C. Kong, Y. Wang, L. Zhu, R. Xu, G. Deng and P. Zhang (2018). "Activated pulmonary tuberculosis in a patient with melanoma during PD-1 inhibition: a case report." Onco Targets Ther **11**: 7423-7427.
Herbst, R. S., P. Baas, D. W. Kim, E. Felip, J. L. Perez-Gracia, J. Y. Han, J. Molina, J. H. Kim, C. D. Arvis, M. J. Ahn, M. Majem, M. J. Fidler, G. de Castro, Jr., M. Garrido, G. M. Lubiniecki, Y. Shentu, E. Im, M. Dolled-Filhart and E. B. Garon (2016). "Pembrolizumab versus docetaxel for previously treated, PD-L1-positive, advanced non-small-cell lung cancer (KEYNOTE-010): a randomised controlled trial." Lancet **387**(10027): 1540-1550.
Im, Y., J. Lee, S. J. Kim, W. J. Koh, B. W. Jhun and S. H. Lee (2020). "Development of tuberculosis in cancer patients receiving immune checkpoint inhibitors." Respir Med **161**: 105853.
Inthasot, V., M. Bruyneel, I. Muylle and V. Ninane (2020). "Severe pulmonary infections complicating nivolumab treatment for lung cancer: a report of two cases." Acta Clinica Belgica: International Journal of Clinical and Laboratory Medicine **75**(4): 308-310.
Jensen, K. H., G. Persson, A. L. Bondgaard and M. Pøhl (2018). "Development of pulmonary tuberculosis following treatment with anti-PD-1 for non-small cell lung cancer." Acta Oncol **57**(8): 1127-1128.
Kanjanapan, Y. and D. Yip (2020). "Characteristics and risk factors for microbial infections during cancer immune checkpoint therapy." Cancer Med.
Karam, J. D., N. Noel, A. L. Voisin, E. Lanoy, J. M. Michot and O. Lambotte (2020). "Infectious complications in patients treated with immune checkpoint inhibitors." Eur J Cancer **141**: 137-142.
Kim, H., S. Y. Ha, J. Kim, M. Kang and J. Lee (2020). "Severe cytomegalovirus gastritis after pembrolizumab in a patient with melanoma." Curr Oncol **27**(4): e436-e439.
Koksal, A. S., B. Toka, A. T. Eminler, I. Hacibekiroglu, M. I. Uslan and E. Parlak (2017). "HBV-related acute hepatitis due to immune checkpoint inhibitors in a patient with malignant melanoma." Ann Oncol **28**(12): 3103-3104.
Krane, N. A., D. M. Beswick, D. Sauer, K. Detwiller and M. Shindo (2020). "Allergic Fungal Sinusitis Imitating an Aggressive Skull Base Lesion in the Setting of Pembrolizumab Immunotherapy." Ann Otol Rhinol Laryngol: 3489420937728.
Kyi, C., M. D. Hellmann, J. D. Wolchok, P. B. Chapman and M. A. Postow (2014). "Opportunistic infections in patients treated with immunotherapy for cancer." Journal for immunotherapy of cancer **2**: 19-19.
Lake, A. C. (2017). "Hepatitis B reactivation in a long-term nonprogressor due to nivolumab therapy." Aids **31**(15): 2115-2118.
Lankes, K., G. Hundorfean, T. Harrer, A. J. Pommer, A. Agaimy, I. Angelovska, A. Tajmir-Riahi, J. Gohl, G. Schuler, M. F. Neurath, W. Hohenberger and L. Heinzerling (2016). "Anti-TNF-refractory colitis after checkpoint inhibitor therapy: Possible role of CMV-mediated immunopathogenesis." OncoImmunology **5**(6).
Lee, J. J., A. Chan and T. Tang (2016). "Tuberculosis reactivation in a patient receiving anti-programmed death-1 (PD-1) inhibitor for relapsed Hodgkin's lymphoma." Acta Oncol **55**(4): 519-520.
Lee, K. A., H. Shaw, V. Bataille and P. Nathan (2020). "Campylobacteriosis following immunosuppression for immune checkpoint inhibitor-related toxicity." Journal for immunotherapy of cancer **8**(2): e000577.

Lee, P.-C., Y. Chao, M.-H. Chen, K.-H. Lan, I. C. Lee, M.-C. Hou and Y.-H. Huang (2020). "Risk of HBV reactivation in patients with immune checkpoint inhibitor-treated unresectable hepatocellular carcinoma." Journal for immunotherapy of cancer **8**(2): e001072.
Lin, X., T. Lu, S. Li, X. Xie, X. Chen, J. Jiang, Y. Qin, Z. Xie, M. Liu, M. Ouyang, N. Zhong, Y. Song and C. Zhou (2021). "Cytomegalovirus infection as an underestimated trigger for checkpoint inhibitor-related pneumonitis in lung cancer: a retrospective study." Clin Transl Oncol **23**(2): 389-396.
Liu, Z., T. Liu, X. Zhang, X. Si, H. Wang, J. Zhang, H. Huang, X. Sun, J. Wang, M. Wang and L. Zhang (2020). "Opportunistic infections complicating immunotherapy for non-small cell lung cancer." Thoracic Cancer **11**(6): 1689-1694.
Lord, J. D., R. C. Hackman, A. Moklebust, J. A. Thompson, C. S. Higano, D. Chielens, G. Steinbach and G. B. McDonald (2010). "Refractory colitis following anti-CTLA4 antibody therapy: analysis of mucosal FOXP3+ T cells." Digestive diseases and sciences **55**(5): 1396-1405.
Lu, J., R. J. Firpi-Morell, L. H. Dang, J. Lai and X. Liu (2018). "An Unusual Case of Gastritis in One Patient Receiving PD-1 Blocking Therapy: Coexisting Immune-Related Gastritis and Cytomegaloviral Infection." Gastroenterology research **11**(5): 383-387.
Malek, A. E., M. Taremi, A. Spallone, J. J. Alvarez-Cardona and D. P. Kontoyiannis (2020). "Necrotizing soft tissue invasive aspergillosis in a cancer patient treated with immunosupressants due to checkpoint inhibitor-induced hepatitis." J Infect **80**(2): 232-254.
Martinot, M., G. Ahle, I. Petrosyan, C. Martinez, D. M. Gorun, M. Mohseni-Zadeh, S. Fafi-Kremer and M. Tebacher-Alt (2018). "Progressive Multifocal Leukoencephalopathy after Treatment with Nivolumab." Emerging infectious diseases **24**(8): 1594-1596.
Murakami, S., R. Usui, Y. Nakahara, T. Kondo, T. Kato and H. Saito (2020). "Readministration of Pembrolizumab after Treatment of Tuberculosis Activated by Initial Pembrolizumab Therapy." Intern Med.
Oltolini, C., M. Ripa, A. Andolina, E. Brioschi, M. Cilla, G. Petrella, V. Gregorc, B. Castiglioni, C. Tassan Din and P. Scarpellini (2019). "Invasive Pulmonary Aspergillosis Complicated by Carbapenem-Resistant Pseudomonas aeruginosa Infection During Pembrolizumab Immunotherapy for Metastatic Lung Adenocarcinoma: Case Report and Review of the Literature." Mycopathologia **184**(1): 181-185.
Pala, L., F. Conforti, E. Cocorocchio, P. Ferrucci, M. T. De Pas, S. Stucchi, M. Repetto, M. Saponara and P. Queirolo (2021). "Course of Sars-CoV2 Infection in Patients with Cancer Treated with anti-PD-1: A Case Presentation and Review of the Literature." Cancer Invest **39**(1): 9-14.
Pandey, A., S. Ezemenari, M. Liaukovich, I. Richard and A. Boris (2018). "A Rare Case of Pembrolizumab-Induced Reactivation of Hepatitis B." Case reports in oncological medicine **2018**: 5985131-5985131.
Pertejo-Fernandez, A., B. Ricciuti, S. P. Hammond, F. M. Marty, G. Recondo, D. Rangachari, D. B. Costa and M. M. Awad (2020). "Safety and efficacy of immune checkpoint inhibitors in patients with non-small cell lung cancer and hepatitis B or hepatitis C infection." Lung Cancer **145**: 181-185.
Picchi, H., C. Mateus, C. Chouaid, B. Besse, A. Marabelle, J. M. Michot, S. Champiat, A. L. Voisin and O. Lambotte (2018). "Infectious complications associated with the use of immune checkpoint inhibitors in oncology: reactivation of tuberculosis after anti PD-1 treatment." Clin Microbiol Infect **24**(3): 216-218.
Pu, D., L. Yin, Y. Zhou, W. Li, L. Huang, L. Cai and Q. Zhou (2020). "Safety and efficacy of immune checkpoint inhibitors in patients with HBV/HCV infection and advanced-stage cancer: A systematic review." Medicine **99**(5): e19013.
Ragunathan, K., S. Dadana and C.-H. Huang (2017). "Hepatitis B Reactivation After Administration of Pembrolizumab (KEYTRUDA): A Unique Case Report: 2145." Official journal of the American College of Gastroenterology | ACG **112**: S1187-S1188.

Redelman-Sidi, G., O. Michielin, C. Cervera, C. Ribi, J. M. Aguado, M. Fernández-Ruiz and O. Manuel (2018). "ESCMID Study Group for Infections in Compromised Hosts (ESGICH) Consensus Document on the safety of targeted and biological therapies: an infectious diseases perspective (Immune checkpoint inhibitors, cell adhesion inhibitors, sphingosine-1-phosphate receptor modulators and proteasome inhibitors)." Clin Microbiol Infect **24 Suppl 2**(Suppl 2): S95-s107.
Rizvi, N. A., J. Mazieres, D. Planchard, T. E. Stinchcombe, G. K. Dy, S. J. Antonia, L. Horn, H. Lena, E. Minenza, B. Mennecier, G. A. Otterson, L. T. Campos, D. R. Gandara, B. P. Levy, S. G. Nair, G. Zalcman, J. Wolf, P. J. Souquet, E. Baldini, F. Cappuzzo, C. Chouaid, A. Dowlati, R. Sanborn, A. Lopez-Chavez, C. Grohe, R. M. Huber, C. T. Harbison, C. Baudelet, B. J. Lestini and S. S. Ramalingam (2015). "Activity and safety of nivolumab, an anti-PD-1 immune checkpoint inhibitor, for patients with advanced, refractory squamous non-small-cell lung cancer (CheckMate 063): a phase 2, single-arm trial." Lancet Oncol **16**(3): 257-265.
Robert, C., G. V. Long, B. Brady, C. Dutriaux, M. Maio, L. Mortier, J. C. Hassel, P. Rutkowski, C. McNeil, E. Kalinka-Warzocha, K. J. Savage, M. M. Hernberg, C. Lebbé, J. Charles, C. Mihalcioiu, V. Chiarion-Sileni, C. Mauch, F. Cognetti, A. Arance, H. Schmidt, D. Schadendorf, H. Gogas, L. Lundgren-Eriksson, C. Horak, B. Sharkey, I. M. Waxman, V. Atkinson and P. A. Ascierto (2015). "Nivolumab in previously untreated melanoma without BRAF mutation." N Engl J Med **372**(4): 320-330.
Robilotti, E. V., N. E. Babady, P. A. Mead, T. Rolling, R. Perez-Johnston, M. Bernardes, Y. Bogler, M. Caldararo, C. J. Figueroa, M. S. Glickman, A. Joanow, A. Kaltsas, Y. J. Lee, A. Lucca, A. Mariano, S. Morjaria, T. Nawar, G. A. Papanicolaou, J. Predmore, G. Redelman-Sidi, E. Schmidt, S. K. Seo, K. Sepkowitz, M. K. Shah, J. D. Wolchok, T. M. Hohl, Y. Taur and M. Kamboj (2020). "Determinants of COVID-19 disease severity in patients with cancer." Nature Medicine **26**(8): 1218-1223.
Rosenberg, J. E., J. Hoffman-Censits, T. Powles, M. S. van der Heijden, A. V. Balar, A. Necchi, N. Dawson, P. H. O'Donnell, A. Balmanoukian, Y. Loriot, S. Srinivas, M. M. Retz, P. Grivas, R. W. Joseph, M. D. Galsky, M. T. Fleming, D. P. Petrylak, J. L. Perez-Gracia, H. A. Burris, D. Castellano, C. Canil, J. Bellmunt, D. Bajorin, D. Nickles, R. Bourgon, G. M. Frampton, N. Cui, S. Mariathasan, O. Abidoye, G. D. Fine and R. Dreicer (2016). "Atezolizumab in patients with locally advanced and metastatic urothelial carcinoma who have progressed following treatment with platinum-based chemotherapy: a single-arm, multicentre, phase 2 trial." Lancet **387**(10031): 1909-1920.
Saikawa, H., H. Nagashima, T. Maeda and M. Maemondo (2019). "Acute cerebellar ataxia due to Epstein-Barr virus under administration of an immune checkpoint inhibitor." BMJ Case Reports **12**(12): 30.
Sakoh, T., M. Kanzaki, A. Miyamoto, S. Mochizuki, T. Kakumoto, K. Sato, Y. Uesaka and K. Kishi (2019). "Ramsay-Hunt syndrome and subsequent sensory neuropathy as potential immune-related adverse events of nivolumab: a case report." BMC cancer **19**(1): 1220-1220.
Schwarz, M., F. Kocher, D. Niedersuess-Beke, J. Rudzki, M. Hochmair, G. Widmann, W. Hilbe and A. Pircher (2019). "Immunosuppression for Immune Checkpoint-related Toxicity Can Cause Pneumocystis Jirovecii Pneumonia (PJP) in Non-small-cell Lung Cancer (NSCLC): A Report of 2 Cases." Clinical Lung Cancer **20**(3): e247-e250.
Shah, N. J., G. Al-Shbool, M. Blackburn, M. Cook, A. Belouali, S. V. Liu, S. Madhavan, A. R. He, M. B. Atkins, G. T. Gibney and C. Kim (2019). "Safety and efficacy of immune checkpoint inhibitors (ICIs) in cancer patients with HIV, hepatitis B, or hepatitis C viral infection." Journal for ImmunoTherapy of Cancer **7**(1).

Si, S., K. Erickson, N. Evageliou, M. Silverman and L. Kersun (2020). "An Usual Presentation of Pneumocystis jirovecii Pneumonia in a Woman Treated With Immune Checkpoint Inhibitor." J Pediatr Hematol Oncol.

Sirgiovanni, M., C. Hinterleitner, M. Horger, N. B. Atique, U. M. Lauer, L. Zender and M. Hinterleitner (2021). "Long-term remission of small cell lung cancer after reactivation of tuberculosis following immune-checkpoint blockade: A case report." Thoracic Cancer **n/a**(n/a).

Suliman, A. M., S. A. Bek, M. S. Elkhatim, A. A. Husain, A. Y. Mismar, M. Z. S. Eldean, Z. Lengyel, S. Elazzazy, K. I. Rasul and N. E. Omar (2020). "Tuberculosis following programmed cell death receptor-1 (PD-1) inhibitor in a patient with non-small cell lung cancer. Case report and literature review." Cancer Immunol Immunother.

Szabados, B., Y. Abu-Ghanem, M. Grant, J. Choy, A. Bex and T. Powles (2020). "Clinical Characteristics and Outcome for Four SARS-CoV-2-infected Cancer Patients Treated with Immune Checkpoint Inhibitors." European Urology **78**(2): 276-280.

Taima, K., H. Tanaka, M. Itoga, Y. Ishioka, A. Kurose and S. Tasaka (2020). "Destroyed lung due to sustained inflammation after chemoradiotherapy followed by durvalumab." Respirology case reports **8**(5): e00580-e00580.

Takata, S., G. Koh, Y. Han, H. Yoshida, T. Shiroyama, H. Takada, K. Masuhiro, S. Nasu, S. Morita, A. Tanaka, S. Hashimoto, K. Uriu, H. Suzuki, Y. Tamura, N. Okamoto, T. Nagai and T. Hirashima (2019). "Paradoxical response in a patient with non-small cell lung cancer who received nivolumab followed by anti-Mycobacterium tuberculosis agents." J Infect Chemother **25**(1): 54-58.

Tetikkurt, S., F. Taş, F. Emre, Ş. Özsoy and Z. T. Bilece (2018). "Significant Neutrophilic Emperipolesis in Squamous Cell Carcinoma." Case Rep Oncol Med **2018**: 1301562.

Uchida, N., K. Fujita, K. Nakatani and T. Mio (2017). "Acute progression of aspergillosis in a patient with lung cancer receiving nivolumab." Respirology case reports **6**(2): e00289-e00289.

Uslu, U., A. Agaimy, G. Hundorfean, T. Harrer, G. Schuler and L. Heinzerling (2015). "Autoimmune colitis and subsequent CMV-induced hepatitis after treatment with ipilimumab." Journal of Immunotherapy **38**(5): 212-215.

van Eeden, R., B. L. Rapoport, T. Smit and R. Anderson (2019). "Tuberculosis Infection in a Patient Treated With Nivolumab for Non-small Cell Lung Cancer: Case Report and Literature Review." Frontiers in Oncology **9 (no pagination)**.

Zhai, M. and S. Zhang (2020). "A Nasopharyngeal Carcinoma Patient With COVID-19 Infection After Immunotherapy: A Case Report and Literature Review." In Vivo **34**(6): 3753-3756.

Zhang, X., Y. Zhou, C. Chen, W. Fang, X. Cai, X. Zhang, M. Zhao, B. Zhang, W. Jiang, Z. Lin, Y. Ma, Y. Yang, Y. Huang, H. Zhao, R. Xu, S. Hong and L. Zhang (2019). "Hepatitis B virus reactivation in cancer patients with positive Hepatitis B surface antigen undergoing PD-1 inhibition." Journal for immunotherapy of cancer **7**(1): 322-322.

Zhou, C., Y. Klionsky, M. E. Treasure and D. S. Bruno (2019). "Pembrolizumab-Induced Immune-Mediated Colitis in a Patient with Concurrent Clostridium Difficile Infection." Case Reports in Oncology **12**(1): 164-170.
